# An Optoelectronic Synapse Based on Two‐Dimensional Violet Phosphorus Heterostructure

**DOI:** 10.1002/advs.202301851

**Published:** 2023-05-25

**Authors:** Xiaoxian Liu, Shuiyuan Wang, Ziye Di, Haoqi Wu, Chunsen Liu, Peng Zhou

**Affiliations:** ^1^ Shanghai Key Lab for Future Computing Hardware and System School of Microelectronics Fudan University Shanghai 200433 China; ^2^ Frontier Institute of Chip and System & Qizhi Institute Fudan University Shanghai 200433 China

**Keywords:** 2D materials, dynamic range, multi‐states, optoelectronic synapse, violet phosphorus

## Abstract

Neuromorphic computing can efficiently handle data‐intensive tasks and address the redundant interaction required by von Neumann architectures. Synaptic devices are essential components for neuromorphic computation. 2D phosphorene, such as violet phosphorene, show great potential in optoelectronics due to their strong light‐matter interactions, while current research is mainly focused on synthesis and characterization, its application in photoelectric devices is vacant. Here, the authors combined violet phosphorene and molybdenum disulfide to demonstrate an optoelectronic synapse with a light‐to‐dark ratio of 10^6^, benefiting from a significant threshold shift due to charge transfer and trapping in the heterostructure. Remarkable synaptic properties are demonstrated, including a dynamic range (*DR*) of > 60 dB, 128 (7‐bit) distinguishable conductance states, electro‐optical dependent plasticity, short‐term paired‐pulse facilitation, and long‐term potentiation/depression. Thanks to the excellent *DR* and multi‐states, high‐precision image classification with accuracies of 95.23% and 79.65% is achieved for the MNIST and complex Fashion‐MNIST datasets, which is close to the ideal device (95.47%, 79.95%). This work opens the way for the use of emerging phosphorene in optoelectronics and provides a new strategy for building synaptic devices for high‐precision neuromorphic computing.

## Graphical abstract

A synaptic device with violet phosphorusheterostructure, a new stable 2D phosphorus is demonstrated, which shows a remarkable light‐to‐dark ratio of over 10^6^, 128 conductance states, and excellent synaptic behaviors.The effect of large dynamic ranges and multi‐states on recognition tasks of varying complexity is further validated, providing guidance for advancingneuromorphic computing applications.

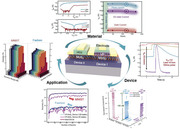

## 1 Introduction

Emerging artificial intelligence applications such as image recognition, motion detection, and autonomous driving require processing massive amounts of data, which poses a serious challenge to the von Neumann architecture that separates memory from computation.^[^ ^1^, ^2^ ^]^ Brain‐inspired neuromorphic computing offers advantages in terms of energy efficiency and operational latency, bringing a promising approach to this challenge.^[^ ^3^ ^]^ In the neuromorphic system, synaptic devices are considered the core units and their properties are essential to achieve efficient, high‐precision computation.^[^ ^4^ ^]^ Due to their attractive characteristics, 2D materials represented by molybdenum disulfide (MoS_2_) appear to be promising candidates to build synapse devices, as their atomic‐scale thickness could reduce operation voltage and energy consumption.^[^ ^5^, ^6^ ^]^ In addition, its tunable bandgap and photo‐electrostatic control ability make it a promising candidate for photoelectric devices. 2D phosphorene such as black phosphorus have also been introduced to construct optoelectronic synapses for the large resonant light absorption (> 20%)^[^ ^7^ ^]^ and better electrostatics, but their stability is still a problem.^[^ ^8^, ^9^ ^]^ Violet phosphorus (VP), considered to be the stable allotrope of phosphorene, has recently been successfully synthesized.^[^ ^10^, ^11^, ^12^, ^13^, ^14^, ^15^ ^]^ With excellent optoelectronic properties, including a direct bandgap of ≈2.54 eV,^[^ ^10^ ^]^ extremely low dark current, and strong light‐matter interactions, VP becomes a potential alternative for optoelectronics. Despite the great breakthroughs in the synthesis and characterization of VP in recent years, its application in photoelectric devices has remained vacant.

Here, by combining the photoelectric potentials of MoS_2_ and VP, we have built a heterostructure synapse and tried for the first time the application of VP in optoelectronic devices. The device exhibits an ultra‐high dark‐to‐light ratio (> 10^6^) due to the relatively large bandgap and strong light‐matter interactions of VP. The optoelectronic coupling and charge transfer within VP‐MoS_2_ heterostructure leads to a strong threshold shift effect when weak light is applied. Thanks to the high light‐to‐dark ratio, the VP‐MoS_2_ heterostructure synapse shows a dynamic range (DR) of over 60 dB and distinguishable conductance multi‐states of 128 (7 bit) at a light intensity of ≈8 pW µm^−2^. Moreover, we demonstrated bionic synaptic behaviors with the heterostructure device, including short‐term and long‐term potentiation (LTP) using light stimulation and long‐term depression (LTD) using electric stimulation. Our heterostructure device owns ultralow off‐state (dark) current in synaptic behaviors, which could minimize the deviation in conductance‐weight mapping (for the lowest but not zero conductance state in the device is mapped into the absolute zero weight in the algorithm, thus bringing deviation between device conductance and algorithm weight in every following states), resulting in improvement in learning accuracy. Using VP‐MoS_2_ device conductance mapping, we simulated MNIST handwriting digits and complicated Fashion‐MNIST image classification tasks to evaluate the impact of DR and multi‐states on accuracy. The accuracy was able to reach 95.23% and 79.65%, respectively, which shows negligible error (0.24%, 0.3%) compared to the ideal device (95.47%, 79.95%). This work fills the device application gap of VP and provides a feasible strategy for building high‐performance synapses for neuromorphic computing.

## 2 Results and Discussion

**Figure 1:**
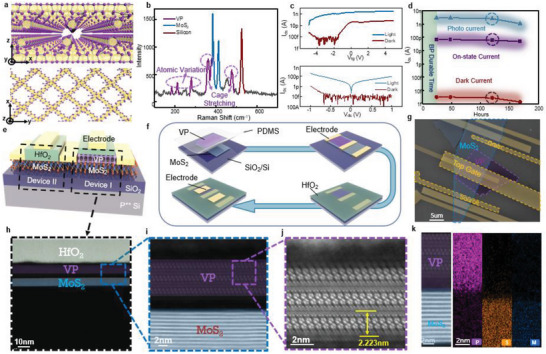
Structure and characterization of VP. a) Schematic of the crystal structure of VP. The upper panel and lower panel represent the cross‐section and top view of the VP lattice, respectively. The violet spheres represent phosphorus atoms while gold beams indicate the configuration of atoms. b) Raman spectrum of the heterostructure device with excitation laser of 532 nm. The blue, violet, and red curves represent the Raman peak of MoS_2_ (383 and 410 cm^−1^), VP (183, 211, 278, 361, 379, and 476 cm^−1^), and silicon (525 cm^−1^) respectively. VP owns a complex Raman spectrum mainly because of its complex lattice structure. c) Transfer and output curves of a common VP phototransistor in darkness and under illumination. d) The current degeneration of a VP phototransistor over 200 h. e) Schematic of the synaptic devices, which consists of device I (right, VP‐MoS_2_ heterostructure) and device II (left, MoS_2_). f) Fabrication flow of the synaptic device, including transferring, source/drain forming, top dielectric deposition, and top gate forming. g) SEM image of the synaptic device. Scale bar: 5 µm. h–j) HAADF STEM image of the synaptic device under different magnifications. The STEM image shows a clean van der Waals interface and lattice structure consistent with the schematic. k) Cross‐sectional STEM image of the VP‐MoS_2_ heterostructure with corresponding EDS mapping.

Progress has been made in the synthesis of 2D VP sheets,^[^ ^10^ ^]^ and the mainstream growth method of 2D VP film is chemical vapor transport with amorphous red phosphorus as the phosphorus source and Sn + SnI_4_ as the transport agents. A thin layer of VP sheets can then be obtained by mechanical or liquid‐phase exfoliation. The lattice structure of VP is described in **Figure** **1**a, which appears to be monoclinic with a space group of P_2/n_, with crystallographic lattice constants of *a* = 9.210 Å, *b* = 9.128 Å, *c* = 21.893 Å, and *β* = 97.776°.^[^ ^10^ ^]^ The VP structure generally shows a bi‐tubular structure with one layer stacked on another vertically along the *z*‐direction, which could be easily exfoliated, as is shown in Figure 1a lower panel. The Raman spectrum of a VP‐MoS_2_ heterostructure excited by a 532 nm laser is shown in Figure 1b. The Raman shift peaks at 183, 211, and 278 cm^−1^ represent the variation modes of VP atoms while the peaks at 361, 379, and 476 cm^−1^ represent the stretching of the atom cages as a whole. The complex Raman spectrum indicates a large density of photon states in VP, which further contributes to the strong light‐matter interaction and electron‐phonon scattering.^[^ ^16^ ^]^ In addition, the few‐layered VP possesses a direct bandgap of ≈2.54 eV^[^ ^10^ ^]^ and usually behaves as an n‐type semiconductor, which indicates its potential to build optoelectronic devices. On this basis, we built VP photo‐transistors and measured their characteristics (VP purchased from Jiangsu XFNANO Materials Tech Co., Ltd, XF282), as is shown in Figure 1c,d. Due to its unique lattice and bandgap characteristics, VP‐based photodevice shows an extremely low dark current (≈fA) and a high light‐to‐dark ratio (≈10^5^). In addition, VP phototransistors were exposed to the ambient air without any encapsulation and could still exhibit negligible degradation of on/off ratio and light‐to‐dark ratio after 200 h, while black phosphorous would undergo performance degradation within 40 h,^[^ ^17^ ^]^ indicating VP as the stable phosphorus (Figure 1d, see Figure S1, Supporting Information for origin data). Interestingly, we found that by forming a heterostructure with MoS_2_, VP could realize its full potential for optoelectronic devices. The heterostructure device could show a larger light‐to‐dark ratio with cumulative current_,_ which could make up for the instantaneous photo‐response of VP phototransistors (see Figure S2, Supporting Information), and make it suitable for mimicking synaptic behaviors. The device schematic and process flow of our heterostructure device is shown in Figure 1e,f, respectively. MoS_2_ and VP were successively transferred to a 300 nm‐thick silicon oxide substrate to form the vertically stacked heterostructure (device I). Electron beam lithography was used to form the electrode pattern and electron beam evaporation (EBE) deposits the source and drain electrodes. Top gate dielectric (30 nm HfO_2_) was then deposited using atomic layered deposition (ALD) with 1 nm seed layer (SiO_2_) using EBE, followed by top gate electrode deposition. Another MoS_2_ device (device II) without VP was built using the same material as the control. The VP stacked here on the top of MoS_2_ works as a photogate, and the thickness of the dielectric and top gate have been carefully determined to ensure the transmission of light. Figure 1g shows the scanning electron microscope (SEM) image of the heterostructure synaptic device. The high‐resolution scanning transmission electron microscope (STEM) was used to characterize the device's microstructure. Figure 1h,j shows the cross‐section HAADF STEM images of the VP under different magnifications, which implies a layered van der Waals structure and is consistent with the lattice in Figure 1a. The STEM image in Figure 1j shows a single‐layer thickness of ≈2.2 nm, which is consistent with the theoretical value.^[^ ^10^ ^]^ Figure 1k shows the STEM image of the VP‐MoS_2_ heterostructure and the corresponding energy dispersive spectroscope (EDS) mapping, which exhibits a clean van der Waals interface between VP and MoS_2_ layers. The heterostructure region was further investigated using an atomic force microscope (AFM), which is provided in Figure S3, Supporting Information. The thickness of VP and MoS_2_ is measured by AFM to be ≈6.66 and 5.85 nm, corresponding to 3 and 9 layers, respectively.

**Figure 2:**
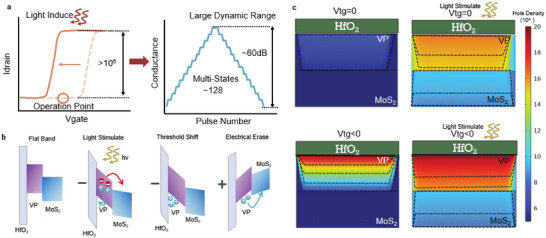
Energy band structure and working mechanism of the synaptic device. a) Schematic of the strategy to obtain large DR. When light stimulation is applied, a threshold shift is induced, creating a “threshold window”. By selecting the operation point within this window, a light‐to‐dark ratio of over 10^6^ could be achieved with weak light, leading to a large DR of over 60 dB and ≈128 conductance states. b) Schematic of the energy bands in different states during the optical potentiation and electrical depression process. When a negative voltage is applied on the top gate, a potential well is formed at the interface of VP and MoS_2_. Then electron‐hole pairs are generated by light stimulation and as the holes are trapped by the potential well, the threshold voltage drifts negatively. c) Hole distribution under different conditions in the heterostructure based on simulation. The dashed lines show the isoline of the hole density.

The general operating principle of our VP‐MoS_2_ synaptic device is described in **Figure** **2**a. When a voltage is applied to the top gate, the device exhibits an n‐type on/off switch, as shown by the dashed line in Figure 2a left panel. Subsequently, when a weak light stimulus is applied, a negative threshold shift of several V can be observed, leading to a “threshold window”, which is shown by the solid line in Figure 2a left panel. Different from the ordinary photocurrent effect where the off‐state current increase obviously under illumination, here the photostimulation mainly act as a trigger to switch the on/off state instead of increasing the off‐state current. By carefully selecting the operation point, the former off‐state can be transformed to the on‐state by light induction, resulting in a large light‐to‐dark DR, which could contain a large number of conductance states, as shown in Figure 2a right panel. It is worth mentioning that the light‐to‐dark ratio is measured and used to describe the binary on‐off switch characteristics (defined by *I_light_*/*I_dark_*), while dynamic range is measured and used in the analogue process (defined by *A_n_*/*A_0_*). In addition, the operation point is typically negative, which means that our device could maintain a high response at a lower off‐state current, leading to improvement in DR. To further investigate the principle of threshold shift, we construct an energy band model for the heterostructure device (device I), as shown in Figure 2b. The theoretical bandgaps of MoS_2_ and VP are about 1.9 eV^[^ ^18^ ^]^ and 2.54 eV,^[^ ^10^ ^]^ respectively. When stacked together without external voltage (flat band), VP and MoS_2_ form a type‐II heterostructure, with the bottom of the conductance band of MoS_2_ higher than VP and the top of the valence band lower than VP. When a negative voltage is applied to the top gate, the device changes from the flat band to the programmed state. Photostimulation is then applied to the device in the programmed state to induce photogenerated electron‐hole pairs. According to the energy band structure, electrons and holes move separately: holes move toward VP and are trapped by the potential well at the interface of VP and HfO_2_ (the properties of VP‐MoS_2_ heterostructure device without the top gate dielectric are shown in Figure S4, Supporting Information, where the separated photogenerated holes are not efficiently trapped, making the threshold shift rather weak and leading to a limited DR); while electrons move toward MoS_2_, leading to an increase in transient channel current. Upon withdrawal of the light stimulus, the trapped holes contribute to the conductivity of the channel, thus resulting in a negative threshold shift. Through applying a positive voltage to the top gate, the band structure changes, leading to a detrapping process of the trapped holes. As a result, the device is reset to the initial state. In this way, the heterostructure device shows the coexistence of optical potentiation and electrical inhibiting, which exactly mimic biological excitatory and inhibitory plasticity and can be mapped to artificial neural networks for neuromorphic computing. As a comparison, we construct another energy band model without VP (device II) to further investigate the role played by VP in such a process, see Figure S5, Supporting Information. Without the strong light‐matter interaction between VP, MoS_2_ produces limited electron‐hole pairs. More importantly, in this case, the separation of electrons and holes cannot be captured efficiently, only a transient photocurrent exists without a significant threshold change. According to our bandgap model, the threshold shift phenomenon is universal in type‐II heterostructure devices and has been observed in other research as well.^[^ ^19^, ^20^ ^]^ However, the VP‐MoS_2_ device still shows advantages in light‐to‐dark ratio due to the large bandgap and strong light‐matter interaction. To confirm the validity of our energy band model, we further use COMSOL Multiphysics to conduct finite element simulation based on the above analysis. More details about the simulation could be found in Section S6, Supporting Information. Figure 2c depicts the distribution of holes in the heterostructure device under different circumstances. When a positive voltage is applied at the top gate without light stimulation, the heterostructure device exhibits an on‐current state with low hole densities, for both VP and MoS_2_ are n‐type semiconductors. When the light stimulation was applied to the device, electron‐hole pairs are generated and separated, with holes moving towards VP and trapped, resulting in the distribution shown in Figure 2c upper panel. In this case, although the concentration of carriers increases overall, the current does not change much since the device is still in on‐state. However, when a negative voltage is applied, the heterostructure device is set to the off‐state. Under such conditions, when light stimulation is applied, the accumulation of holes in VP leads to threshold voltage shift thus turning the device from the non‐conducting state into conducting state, leading to a large DR through light stimulation, as shown in Figure 2c lower panel.

**Figure 3:**
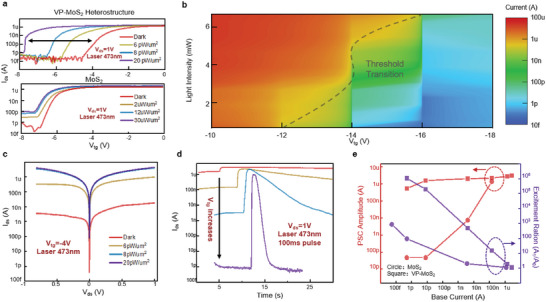
Threshold drift and optoelectronic characteristics of the VP‐MoS_2_ synaptic device. a) Transfer curve of the two structured devices under different laser illuminations. *V_ds_* is fixed at 1 V and the laser wavelength is 473 nm. The heterostructure device shows a considerable threshold shift compared to the MoS_2_ transistor. b) Current mapping under different *V_tg_* and laser intensities. The dashed line indicates the trend of threshold voltage shift. c) Output curve of the synapse device, where *V_tg_* is fixed at −8 V. d) Output waveforms at different *V_tg_*, when a single laser pulse is applied. The PSC can reach ≈1 µA regardless of *V_tg_*. e) Performance comparison between the two structured devices. The VP‐MoS_2_ device could maintain a higher excitement ratio and PSC amplitude than the MoS_2_ device.

Experiments are implemented to verify these analyses. **Figure** **3**a upper panel shows the transfer curve of the VP‐MoS_2_ heterostructure device under varying illuminations, where the source‐drain voltage (*V_ds_*) is fixed at 1 V and the laser wavelength is 473 nm. Obvious threshold shift can be observed for illumination as low as 6 pW µm^−2^ and expand with increasing intensity, which is generally consistent with our simulation. The actual laser intensity may be lower, taking into account the influence of the top gate dielectric and electrodes. As the intensity increases to 20 pW µm^−2^, the threshold voltage has changed from about −4 V to below −8 V, which is desired for our operation. According to the transfer curve, when the top gate voltage (*V_tg_*) is fixed in this range (−4 to −8 V), a 20 pW µm^−2^ optical stimulation could lead to a light‐to‐dark ratio of up to 10^6^. To verify such a conclusion, we chose an operation point of −4 V and test the output curve under varying light intensities, as shown in Figure 3c. At such an operation point, the output current increases to around 1 µA under 20 pW um^−2^ illumination, which is consistent with the transfer curve. In addition, we test the transfer curve of device II as a control, as shown in Figure 3a lower panel. In this device, almost no change in threshold voltage is observed even when 30 µW µm^−2^ of strong light is applied, which is in line with our previous analysis. Such a phenomenon has been repeatedly verified in different batches of devices with this heterostructure (Figure S7, Supporting Information). Although the threshold voltage varies slightly with the thickness of 2D materials, this phenomenon could be reproduced in each device. Next, we stimulate the device with a single laser spike and test its response, as shown in Figure 3d. When it comes to neuromorphic computing, the laser spike here could simulate presynaptic input, and the channel current is monitored as post‐synaptic current (PSC). The stimuli are applied at different operation points (different *V_tg_*). Despite the fact that the base current of the device decreases with increasing *V_tg_*, the PSCs almost all reach the µA level, which is consistent with the on‐state current of our device. As a result, the excitement ratio (PSC/base current) increases rapidly as *V_tg_* increases, and can exceed 10^6^ thanks to the extremely low dark current of VP. Similar tests are carried out using device II (see Figure S9, Supporting Information). A comparison of the two devices is shown in Figure 3e, where the squares represent the performance of the VP‐MoS_2_ heterostructure device (device I) and the circles represent the MoS_2_ transistor (device II). To eliminate the effect of different original threshold voltages, the *x*‐axis has been unified as base current rather than top gate voltage. Regarding the PSC amplitude (left axis), the MoS_2_ transistor shows high PSC amplitude only when the base current itself is relatively high, whereas VP‐MoS_2_ exhibits high PSC amplitude irrespective of the base current. This discrepancy leads to at least a 3‐magnitude improvement in excitement ratio (right axis), representing a strong synaptic response to the stimulus. In addition, to visualize the threshold shift of the VP‐MoS_2_ device, we collected the source‐drain currents for different top gate voltages as well as for different light intensities of another synaptic device and shown by color mapping in Figure 3b (see original curves in Figure S8, Supporting Information). Considering 10 nA as the boundary between on and off switching, the dashed line could reflect the shift in threshold voltage, that is, the threshold changes from −12 to −16 V as the laser intensity increases from 0 to 6 µW µm^−2^, which leaves a “threshold window” of ≈4 V, sufficient for synaptic device operation.

**Figure 4:**
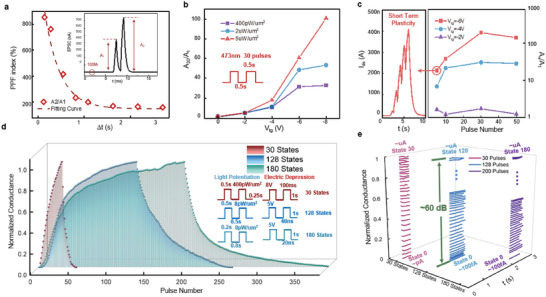
Short‐ and long‐term synaptic plasticity in VP‐MoS_2_ heterostructure device. a) PPF indexes at different intervals. The inserted image shows a typical waveform of the PPF characteristics. The dashed line indicates the fitting curve at the experimental points. b) Variation of PSC gain for different optical intensities and base current (*A_0_*) during 30 pulses of stimulation. The frequency of the laser spikes is fixed at 2 Hz with a 473 nm laser. c) PSC gain as a function of top gate voltage and pulse numbers. The left panel shows a typical waveform of short‐term plasticity, which gradually changes into long‐term plasticity as the number of pulses increases. d) Normalized LTP and LTD with 30128 and ≈180 conductance states. Optical and electrical spikes are applied for the potentiation and depression processes, respectively. The waveforms used are shown in the insert. e) Specific waveforms of every state extracted from(d), which indicates distinguishable and stable conductance states.

We further explored the potential of the VP‐MoS_2_ device for the simulation of synaptic plasticity and behavior. Dual laser pulses with different intervals were applied to the device to test the paired‐pulse facilitation (PPF) characteristics, which is essential to simulate biologically typical short‐term plasticity (STP). The inset in **Figure** **4**a shows a typical output waveform, where the base current is around 100 fA, and *A_1_* and *A_2_* represent the amplitudes after the first and second laser pulses. The second pulse exhibits a stronger response than the first pulse and quickly recovers to the base current, showing typical PPF characteristics. Waveforms based on different interval times are shown in Figure S10, Supporting Information. When the interval is as low as 150 ms, our device shows a fairly high PPF index (determined by *A_2_*/*A_1_*) of up to 853% and gradually recovers to 100% as the interval is increased above 3000 ms. The dashed line here indicates the fitting curve at the experimental points, which obeys an exponential decay, consistent with the theoretical result.^[^ ^21^ ^]^ Moreover, the long‐term plasticity of the heterostructure synaptic device was explored by increasing the number of laser pulses. As shown in Figure 4b, 30 laser spikes with different intensities (0.4, 2, 6 µW µm^−2^) were applied to achieve a progressive excitatory PSC modulation, which simulated the LTP plasticity. The PSC gain (determined as *A_30_*/*A_1_*) increases significantly as the base current A0 decreases (increase in *V_tg_*) as well as the laser power increases. Figure 4c shows the PSC gain (*A_n_*/*A_1_*) as a function of top gate voltage and pulse number. When the pulse number is as low as 5, the synapse device generally exhibits STP, as shown in Figure 4c left panel. In this case, the PSC increases linearly with the accumulation of laser spikes and falls back to the base current a few seconds after the removal of the laser. The device shows a clear transition from STP to LTP as the pulse number increases, with an increase in PSC gain and retention time (Figures S11 and S12, Supporting Information). In addition, a higher top gate voltage will also significantly increase the PSC gain (Figure S12, Supporting Information). However, the extremely high DR is achieved at the expense of linearity and retention time, which are also critical for neuromorphic computing. To balance all these key metrics, the operation point was carefully selected to achieve a large DR (60 dB) with 30 conductance states and fair linearity (1.31), as is shown in Figure 4d red curve (more details in Figure S13, Supporting Information), which could meet the requirements for high‐precision neuromorphic computing.^[^ ^4^ ^]^ A large DR means the potential to contain more conductance states: Considering a synaptic device with linear conductance change, the theoretical maximum number of conductance states N could be approximately estimated by the following formula.

(1) $$N=\frac{A_{max}-A_{min}}{A_{1}-A_{min}}\;=\frac{\frac{A_{max}}{A_{min}}-1}{\frac{A_{1}}{A_{min}}-1}\;=\frac{A_{min}\left(DR-1\right)}{R}$$

where *A_max_*, *A_min_*, and *A_1_* represent the maximum state, minimum state, and the state after one pulse, separately. *DR* represents the dynamic range and *R* for the resolution (the minimum stable and distinguishable gap between adjacent states) of the synaptic device. For the resolution depends on the noise level which is hard to improve, while *A_min_* depends on the leakage current of the synaptic device which is not convenient to be so high, improving *DR* is a reliable and efficient strategy to get multi‐conductance states. To further investigate the capability of the heterostructure device to obtain distinguishable conductance multi‐states, we applied more pulses with smaller intensity for both potentiation and depression. Figure 4d shows the normalized long‐term synaptic plasticity using optical pulses for potentiation and electrical pulses for depression under different circumstances. By applying 128 light pulses (8 pW µm^−2^, 0.5 s) for stimulation, 128 (7‐bit) stable, non‐crossing, and distinguishable conductance states are generated. The original waveforms are shown in Figure S14, Supporting Information, with a base state of around 100 fA, and after 128 pulses of stimulation, the current rises to ≈µA, indicating a strong capability to map synaptic device conductance to neural network weights. In addition, by applying over 200 pulses (8 pW µm^−2^, 0.2 s), a maximum of ≈180 conductance states can be obtained after excluding the crossed states (original waveforms shown in Figure S15, Supporting Information). Figure 4e depicts the waveforms of each state extracted from Figure 4d, which are distinguishable and stable. However, limited by the instability of light stimulation and strong light response of the device, state intervals smaller than ≈nA hardly exist stably, limiting the further increase in the number of states. In addition, the energy consumption of our device has also been calculated in these three different conditions (See Figure S19, Supporting Information). Our device shows low energy consumption as low as 0.8 pJ per spike, which shows the energy superiority of synaptic devices. The proposed VP‐MoS_2_ heterostructure synapse generally exhibits a high light‐to‐dark DR of over 60 dB, as well as 128 (7‐bit) distinguishable conductance multi‐sates, providing a new strategy for improving the DR and multi‐states in synaptic devices.

**Figure 5:**
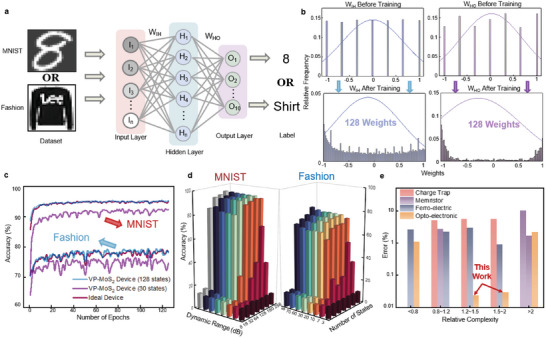
Image classification of MNIST and Fashion‐MNIST datasets using VP‐MoS_2_ synaptic devices. a) Schematic of the neural network model used for image classification simulation. b) Simulated weight distributions before and after training, including *W_IH_* (40 000 synapses) and *W_HO_* (1000 synapses). c) The learning accuracy as a function of training epochs for both datasets. The red, blue, and violet lines represent the ideal device and the large‐dynamic‐range VP‐MoS_2_ device with 128 and 30 conductance states, respectively. The heterostructure device shows negligible distinction from the ideal device in both classification tasks. d) Dependence of classification accuracy on different *DRs* and state numbers. *DR* and state numbers affect learning accuracy over a wide range, especially when the *DR* is below 20 dB, where accuracy is severely degraded. The infinity *DR* is achieved by setting the off‐state current to 0 (ideal condition). e) The learning error of synaptic devices in classification tasks with different complexity. The dataset complexity is defined as the average information entropy of images. For tasks with higher complexity, synaptic devices generally show a larger error rate, while our device could maintain a lower error rate in different tasks.

Finally, we used the NeuroSim multilayer perceptron (MLP) neural network simulator^[^ ^22^ ^]^ to validate the ability of the VP‐MoS_2_ synaptic device to perform image classification tasks. The neural network used is shown in **Figure** **5**a, which consists of an input layer, a hidden layer, and an output layer. Each neuron node in one layer is connected to each node in the following layer, forming a fully connected neural network. Neuron nodes are connected via synaptic devices, and device conductance represents network weights. *W_IH_* and *W_HO_* represents the weight matrix between the input and hidden layers and between the hidden and output layers, respectively. We have made the necessary modifications to the original network model in the NeuroSim simulator to make it suitable for our classification tasks. We performed the image classification based on two standard datasets: MNIST and Fashion‐MNIST (an MNIST‐like dataset with higher complexity).^[^ ^23^ ^]^ The input image data has been pre‐processed into grayscale data of each pixel as the input layer (20 × 20 for MNIST and 28 × 28 for Fashion). The network contains 100 hidden neurons and 10 output neurons (referring to 10 kinds of labels, i.e., handwriting digits or objects), more details about the simulation could be found in Sections S16–20, Supporting Information. Figure 5b shows the distribution of weights before and after MNIST training, consisting of *W_IH_* and *W_HO_*. The initial weights for both matrices are set randomly from seven states (0, ±1, ±0.33, and ±0.66), and the weights after training are mapped to 128 states, corresponding to the 128 conductance states of VP‐MoS_2_ device, indicating the update of the network weights.

The classification accuracy as a function of training epochs is shown in Figure 5c. According to the simulation, our device eventually reach an accuracy of 95.23% for MNIST and 79.65% for the Fashion dataset, which is close to those of ideal devices (95.47% and 79.95%). The original algorithm of the simulator is designed specifically for the 20 × 20 MNIST dataset, the learning accuracy for the Fashion dataset is relatively lower but still could reflect the superiority of our device by calculating its error with the ideal device, which could exclude the influence of the algorithm. The detailed parameters and results of our simulation could be founded in Table S2, Supporting Information. It is worth mentioning that in the actual classification process, the conductance states of real devices are mapped into the synaptic weights in the algorithm, where the lowest conductance state is transferred into weight 0. However, due to the physical limitation of the device, the absolute 0 weight is unreachable, which would affect all the conductance‐weight mapping and finally lead to accuracy deviation between the ideal condition and the real device.^[^ ^24^ ^]^ Our heterostructure synaptic device owns an ultralow off‐state current that minimizes such deviation, resulting in an accuracy comparable to the ideal cases. We further investigated the dependence of classification accuracy on DR. Figure 5d shows the final classification accuracy for different *DR* and the number of conductance states while keeping other parameters constant (consistent with the previous simulation for device I). In these cases, the change in *DR* occurs with a decrease in the off‐state while keeping the on‐state constant. Simulation results show that for simple classification tasks like MNIST, accuracy is strongly suppressed when the *DR* is below 10 dB, and for *DR* over 20 dB, the increase of *DR* could still lead to improvement in learning accuracy. In addition, the increase in state number will also improve learning accuracy significantly. As for classification tasks with higher complexity like Fashion‐MNIST, such dependence becomes more pronounced. In Figure 5e, we further investigated the learning performance of different synaptic devices for image classification tasks with different complexity. The *x*‐axis shows the complexity of different datasets, and the *y*‐axis shows the error between ideal cases (software implementation) and real devices. The complexity of a dataset is defined as the average information entropy of the images:

(2) $$H\left(U\right)=-\Sigma p_{i}\cdot lg\left(p_{i}\right)$$

Where *p_i_* represents the distribution frequency of a certain grayscale (or RGB) value in the image (See Section S19, Supporting Information for the detailed calculation method). In those cases, most synaptic devices show relatively high error (over 2%) due to low DR and fewer state numbers.^[^ ^25^, ^26^, ^27^, ^28^, ^29^, ^30^, ^31^, ^32^, ^33^, ^34^ ^]^ By combining large DR and sufficient state numbers, our device shows negligible error from ideal cases, even in relatively complex tasks like Fashion‐MNIST. This suggests that for future intelligent scenarios, increasing the DR and state numbers of synaptic devices is necessary and urgent.

## 3 Conclusion

In this work, we have achieved the first device application of the stable phosphene VP and proposed the VP‐MoS_2_ heterostructure for optoelectronic synapse. VP exhibits extremely low dark currents and high dark‐to‐light ratios and restricts photogenerated holes by forming the type‐II heterostructure with MoS_2_, leading to a strong shift in threshold voltage. This phenomenon was exploited to realize optically induced synaptic plasticities with large DR (≈60 dB) and conductance states (128, 7‐bit), which is crucial for neurophormic computing. Furthermore, there is still room for improvement in these indicators. For example, the light‐matter interaction could be enhanced by optimizing the design of the top gate dielectric. And the retention time is limited by the charge‐trapping capability of the interface, which can be further improved by introducing additional physical fields. We further explored the potential of the VP‐MoS_2_ synaptic device for MNIST and Fashion‐MNIST image classification through the NeuroSim simulator and achieved accuracies comparable to that of ideal devices. The results indicate the significance of large DR and multi‐states for accuracy optimization. This work demonstrates the potential of VP as a unique optoelectronic material for synaptic devices and provides a viable strategy for implementing high‐precision neuromorphic computing.

## 4 Experimental Section

### Fabrication of the Heterostructure Synaptic Device

Few‐layer MoS_2_ and VP (Jiangsu XFNANO Materials Tech Co., Ltd, XF282) were transferred to a silicon substrate with 300 nm‐thick SiO_2_, forming the heterostructure. The electrode pattern was defined by electron beam lithography (EBL) and Cr/Au metal was deposited by electron beam evaporation (EBE) to form the source and drain. For the top gate dielectric, 2 nm SiO_2_ was deposited as a seed layer using EBE, followed by atomic layer deposition (ALD) to form 30 nm HfO_2_ as the dielectric layer. Then the top gate electrodes were formed again through EBL and EBE.

### Finite Element Simulation of Heterostructure Device

The simulation process was realized based on CMOSOL Multiphysics. The semiconductor module and electromagnetic waves module were combined to simulate the generation, migration, and trapped process of photo‐generate carriers. The material characteristics of VP and MoS_2_ were set according to former research. The vertical dimension of the device was set to 10 nm, which was negligible compared with a channel length of 3 um. The top gate dielectric (HfO_2_) was placed at the top of VP, where the Shockley–Read–Hall model was used to simulate the trap‐assisted surface recombination process. Through the Newton iteration method, the carrier concentration and corresponding port current were calculated under different voltage and illumination conditions.

### Characterization and Measurement of the Heterostructure Synaptic Device

The surface morphology of the heterostructure device was characterized by AFM, showing the thickness of VP and MoS_2_ are about 6.66 and 5.85 nm, respectively. To examine the constructed van der Waals heterojunction interface, a cross‐sectional analysis was performed using HAADF STEM technology with EDS elements mapping analysis. In addition, the 2D layered materials were characterized by Raman spectroscopy. VP showed strong peaks near 361, 379, and 476 cm^−1^, and MoS_2_ showed peaks at 383, and 410 cm^−1^. The Cascade probe station equipped with a Keysight B1500A semiconductor analyzer was used to characterize the electrical properties of the heterostructure device and the simulation of synaptic plasticity under the ambient environment. The optical stimulation was applied through a 473 nm blue light laser and the corresponding light intensity was measured by a light intensity meter.

### Simulation of Image Classification Based on the Fully Connected Neural Network

The simulation process was realized based on the NeuroSim MLP simulator. A complete epoch included loading the data set, defining a fully connected network model, training the network, testing the network, and calculating the classification accuracy. The training process included feed‐forward and back‐propagation processes. To execute the simulation using the Fashion‐MNIST dataset, the topology of the network had been modified, and the learning rate had been carefully changed to optimize the learning accuracy.

## Conflict of Interest

The authors declare no conflict of interest.

## Author Contributions

X.L. and S.W. contributed equally to this work. X.L. and S.W. co‐wrote the manuscript; Z.D. and H.W. assisted with material characterization and testing; P.Z. conceived the idea and supervised the work. All authors provided suggestions for revisions and improvements to the work.

## Supporting information

Supporting Information

## Data Availability

The data that support the findings of this study are available from the corresponding author upon reasonable request.
