# Supplementary material for: An Optoelectronic Synapse Based on Two‐Dimensional Violet Phosphorus Heterostructure
Source: Adv Sci (Weinh). 2023 May 25;10(22):2301851. doi: 10.1002/advs.202301851 (PMC10401094; doi:10.1002/advs.202301851)
Supplement: Supplementary file 1 — Supporting Information [file ADVS-10-2301851-s001.pdf]

ADVANCED SCIENCE
Open Access

Supporting Information

for *Adv. Sci.*, DOI 10.1002/advs.202301851

An Optoelectronic Synapse Based on Two-Dimensional Violet Phosphorus Heterostructure

Xiaoxian Liu, Shuiyuan Wang, Ziye Di, Haoqi Wu, Chunsen Liu and Peng Zhou**

Supplementary Information

An Optoelectronic Synapse Based on Two-Dimensional Violet Phosphorus Heterostructure

Xiaoxian Liu[1,†], Shuiyuan Wang[1,†,*], Ziye Di[1], Haoqi Wu[1], Chunsen Liu[2], and Peng Zhou[1,*]

[1]Shanghai Key Lab for Future Computing Hardware and System, School of Microelectronics, Fudan University, Shanghai 200433, China

[2]Frontier Institute of Chip and System & Qizhi Institute, Fudan University, Shanghai 200433, China

[†]These authors contributed equally to this work

*Author to whom correspondence should be addressed: sy_wang@fudan.edu.cn, pengzhou@fudan.edu.cn

Table of Contents

Section 1 | Temporal stability of VP transistors 3

Section 2 | Photoelectric response of VP transistors 4

Section 3 | Morphological characterization of the heterostructure device......................... 5

Section 4 | VP-MoS_2 transistors without top dielectric 6

Section 5 | Bandgap model of MoS_2 device.. 7

Section 6 | Finite elements simulation of heterostructure device...................................... 8

Section 7 | Generality of the threshold shift for VP-MoS_2 device 10

Section 8 | Current mapping under varying laser intensities and gate voltages 12

Section 9 | Single pulse response of the VP-MoS_2 and MoS_2 devices 13

Section 10 | PPF characteristics of the VP-MoS_2 synaptic devices 14

Section 11 | Potentiation modulated by gate voltage with 5 laser pulses 15

Section 12 | Potentiation modulated by gate voltage with 30, 50 laser pulses 16

Section 13 | 30 pulses long-term characteristics of VP-MoS_2 heterostructure devices 17

Section 14 | 128 pulses long-term characteristics of VP-MoS_2 heterostructure devices ... 18

Section 15 | 200 pulses long-term characteristics of VP-MoS_2 heterostructure devices ... 19

Section 16 | Electrical excitation process of VP-MoS_2 heterostructure device.................. 21

Section 17 | Definition of distinguishable conductance states 22

Section 18 | Neural network simulation for image classification 23

Section 19 | Conductance linearity of the VP-MoS_2 devices 25

Section 20 | Parameters and results of the simulation 26

Section 21 | Complexity definition of different classification tasks................................. 27

Section 22 | Calculation of synaptic energy consumption... 29

Section 1. Temporal stability of VP transistors

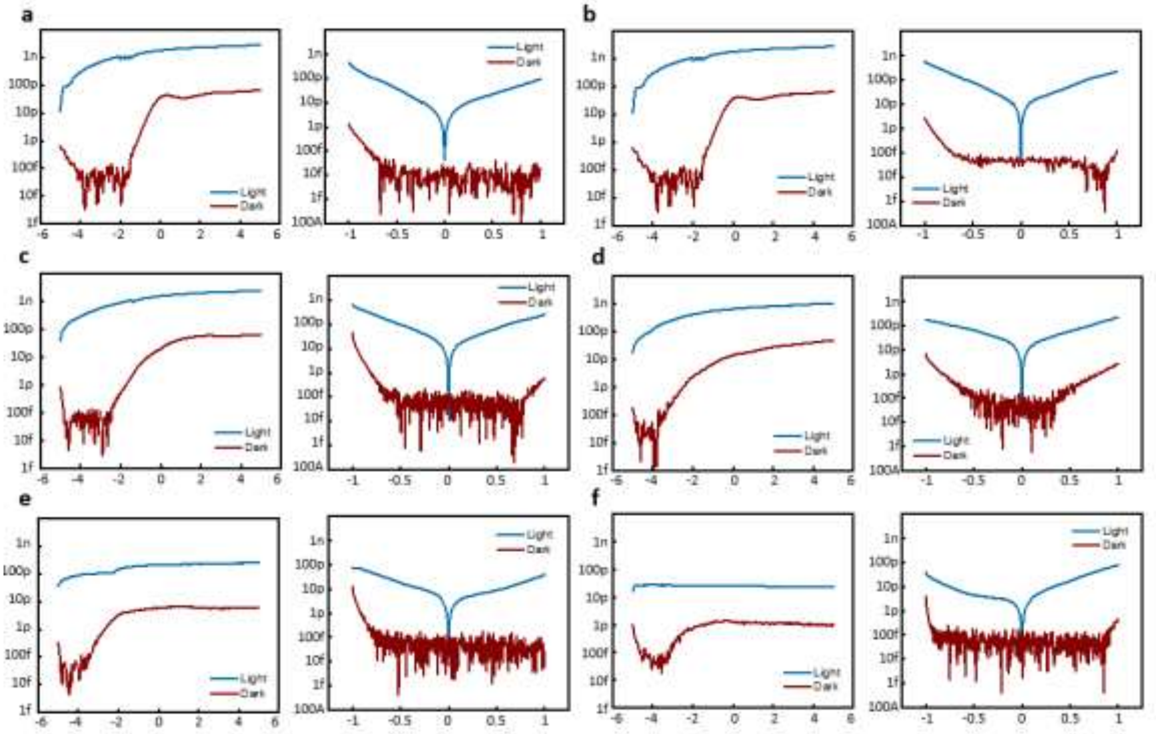

Figure S1 | Time stability of VP transistors. The transfer and output curves of the VP optoelectronic transistor have been tested in 340 hours. **a~f**, the curve on the 1st, 2nd, 3rd, 5th, 7th, $10^{th,}$ and 14th days, respectively. The device can remain stable for at least 10 days when exposed to air without any protection, with a negligible drop in on-state current, confirming that VP is a stable 2D phosphorene material. After 340 hours, the photocurrent did not change much, although the on-state current decreased in the dark, implying that VP shows stable optoelectronic properties.

Section 2. Photoelectric rensponse of VP transistors

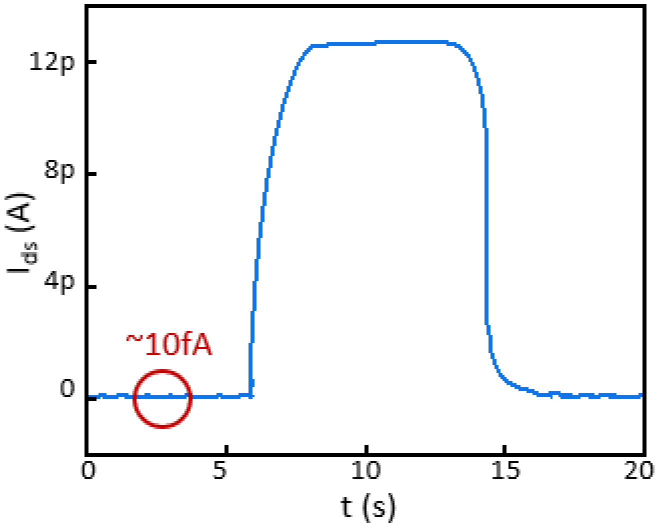

Figure S2 | The optoelectronic characteristics of VP transistors. The VP transistor generally shows an immediate volatile response to illumination (473 nm).

Section 3. Morphological characterization of the heterostructure device

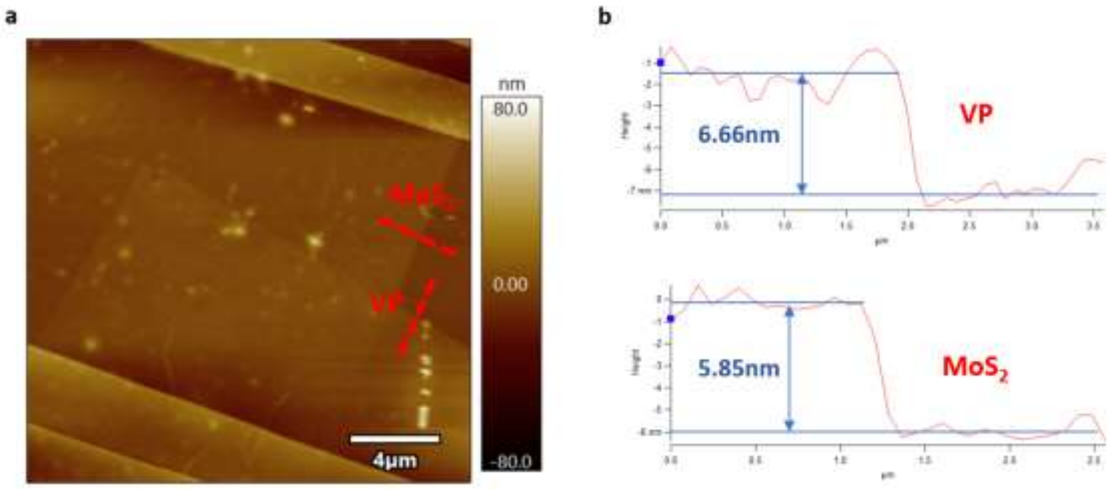

Figure S3 | Atomic force microscope (AFM) characterization of the VP-MoS_2 device. a, The AFM image shows flat and uniform surface of VP-MoS_2 heterostructure. **b**, The thicknesses of MoS_2 and VP are about 5.85 and 6.66 nm, respectively. For the thickness of the monolayer MoS_2 and VP are 0.65 nm and 2.3 nm[1,2], AFM results suggest that the MoS_2 sheet consists of 9 layers and the VP sheet consists of 3 layers.

Section 4. VP-MoS_2 transistors without top dielectric

We also build the heterostructure synaptic device without top gate dielectric to act as a control, where the gate voltage is applied through the electrode on VP. When the top dielectric is removed, the device could still show threshold shift phenomenons under illumination (Supplementary **Figure S4a**). However, without the top gate dielectric, the separated photogenerated holes cannot be effectively trapped, which makes the threshold shift quite weak, resulting in a dynamic range that is not as large as that of the device with top dielectric (Supplementary **Figure S4 b, c**).

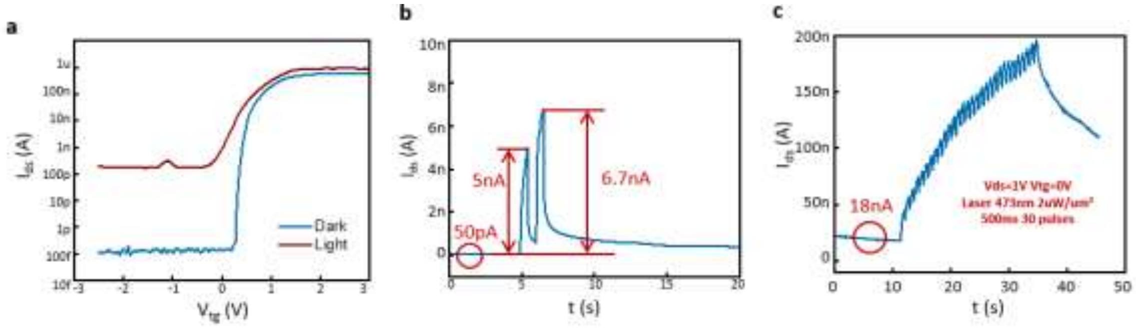

Figure S4 | The characteristics of the VP-MoS_2 heterostructure transistor without top gate dielectric. **a**, Transfer curve of the heterostructure device under dark and illumination (473 nm 2uW/um^2) conditions. **b**, The PPF characteristics of the heterostructure device without top dielectric. **c**, The LTP characteristics with 30 optical pulses.

Section 5. Bandgap model of the MoS_2 device

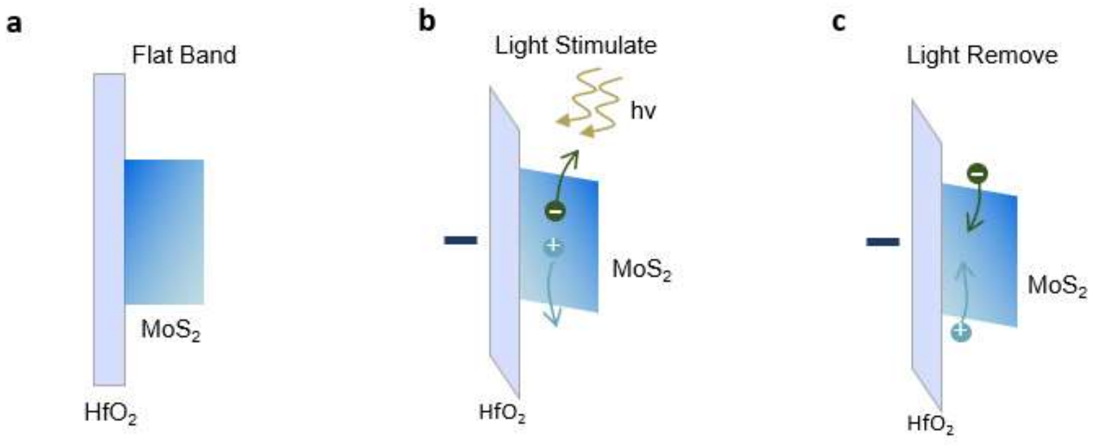

Figure S5 | Bandgap model of the MoS_2 device.

Section 6. Finite elements simulation of the heterostructure device

We used COMSOL Multiphysics to construct a heterostructure model for our synaptic device. In this model, the semiconductor module and electromagnetic wave (frequency domain) module are combined to simulate the photo-generated carrier dynamics. The light beam is applied at the top of VP, traveling through the heterostructure device and generating electron-hole pairs, which move under the effect of the external electric field. **Figure S6ab** shows the electron concentration distribution during this process. When the gate voltage turns negative, the electron density indicates the device at off-state. Then a light beam is applied, and the trapped holes (see **Figure 2c**) turn the device from off-state to on-state, as shown in the Figure. The parameters used in our simulation are shown in **Table S1**.

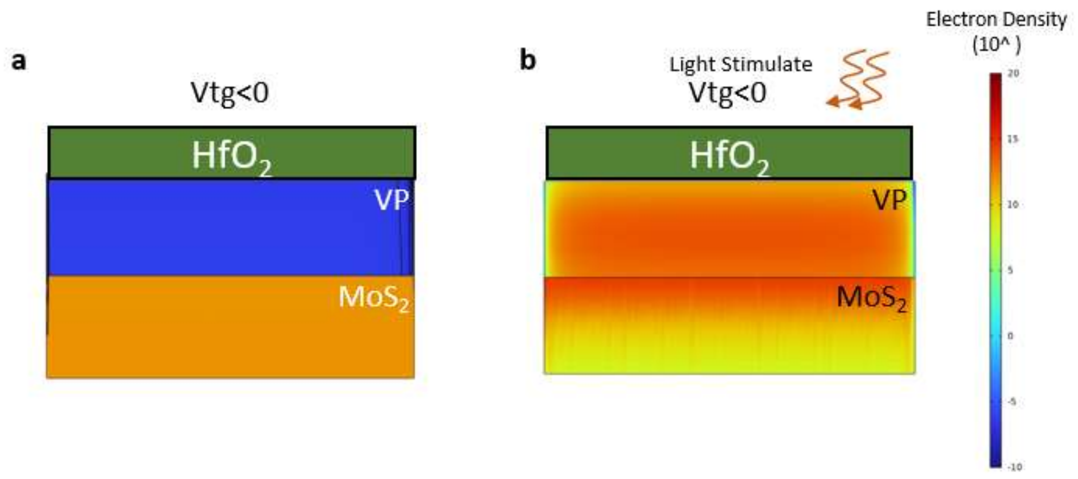

Figure S6 | The simulation results of the VP-MoS_2 heterostructure device. a~b,Electron concentration in the heterostructure device. When the gate voltage is negative, the device could change from nonconducting state to conducting state by applying light stimulation, as shown in the electron concentration mapping.

Parameter	Value	unit
Bandgap	2.5 (VP)/1.9 (MoS_2)	eV
Electron affinity	3.7 (VP)/4.9 (MoS_2)	eV
Mobility	50 (hole)/100 (electron)	$cm^2/(V*s)$
Device dimension	3000*10	nm
Wavelength	473	nm
Laser power	5	uW
Drain voltage	1	V

Table S1 | The parameters used in simulation.

Section 7. The generality of the threshold shift for VP-MoS_2 device

To confirm the generality of the threshold shift, four typical heterostructure devices with different VP thicknesses were tested (Supplementary **Figure S7a-d**). Despite the differences in threshold voltage due to the different thicknesses, threshold shift phenomena can all be observed. In addition, the range of threshold shifts varies due to different carrier capture capabilities, but all of the VP-MoS_2 heterostructure devices show a larger drift window than the MoS_2 transistors.

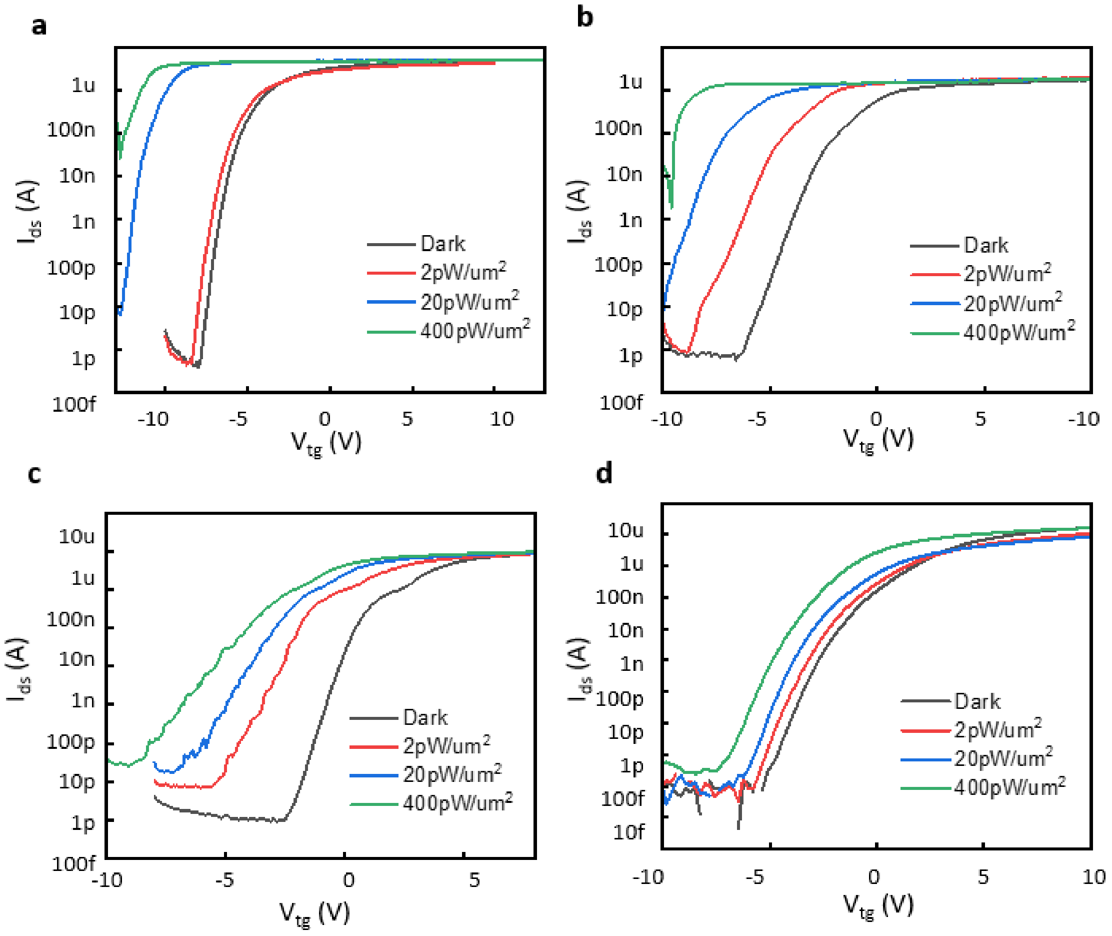

Figure S7 | Transfer curve of heterostructure devices with varying VP thicknesses. The thickness of VP in a, b, c and d are ~8.6, ~8.4, ~4.5 and ~2.2 nm, refers to 4, 4, 2 and 1 layer respectively. The thickness of VP would affect the bandgap as well as optical response. As far as we are concerned,

thicker VP means stronger light-matter interaction, bringing efficient photo-generated pairs, thus leading to better optical response. According to our experiments, thicker VP of 3 or 4 layers could show obviously better threshold shift than 1 or 2 layers. However, VP could not be too thick, for bulk VP owns relatively smaller bandgap, which would cause increases in the dark current.

Section 8. Current mapping under varying laser intensities and gate voltages

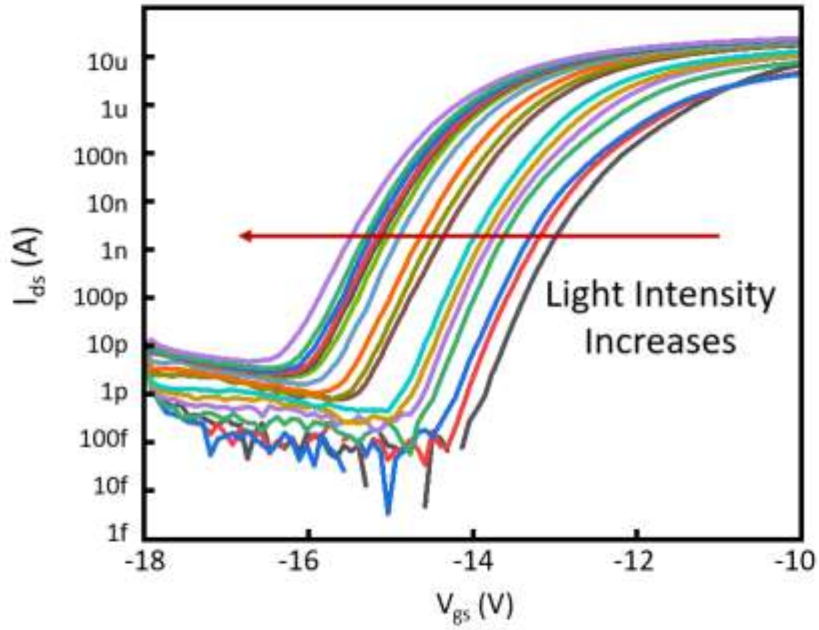

Figure S8 | The current mapping under different light intensities and gate voltages. The figure shows the current under different laser intensities (473 nm, from darkness to 6.5uW/um^2) and gate voltage (from -10 V to -18 V), which are used for the color mapping in Figure 2b in the main text.

Section 9. Single pulse response of the VP-MoS_2 and MoS_2 devices

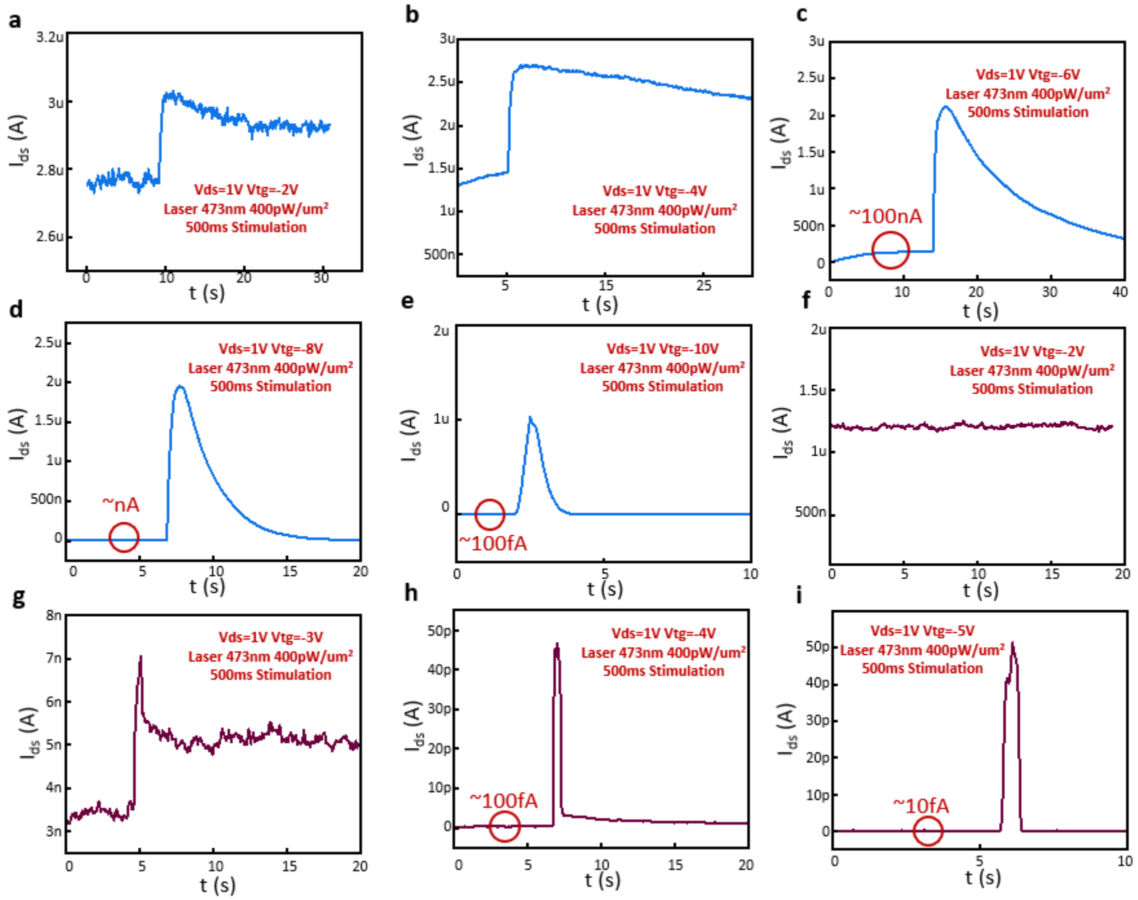

Figure S9 | Single pulse waveform of the VP-MoS_2 and MoS_2 devices under different gate voltages. a~e, Single pulse response of our heterostructure device. **f~i**, Single pulse response of MoS_2 transistor shown in Figure 3d. The test details are shown in the figure insert.

Section 10. PPF characteristics of the VP-MoS_2 synaptic devices

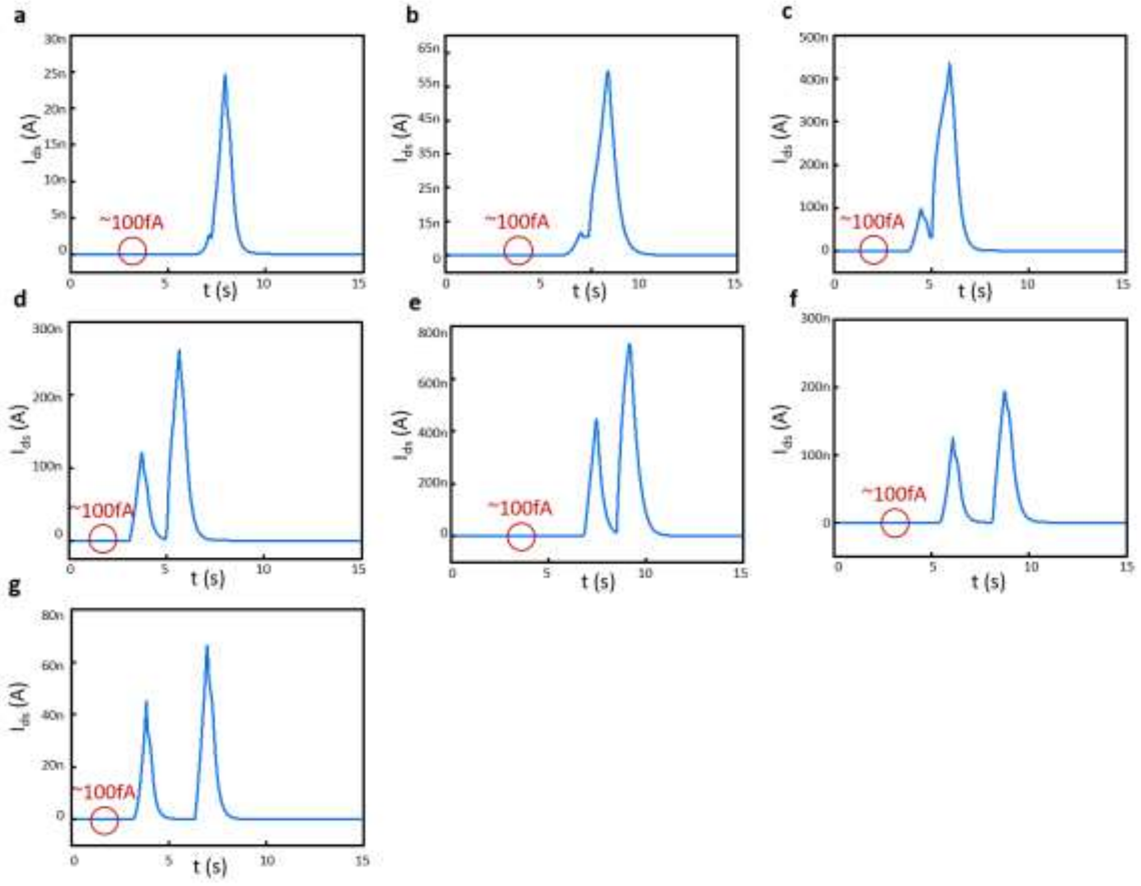

Figure S10 | The PPF characteristics with different intervals. The gate voltage is fixed at -6 V, leading to an off-state current of ~100 fA, and light intensity is fixed at 400 pW/um^2. **a~f**, Interval times of 150, 300, 600, 900, 1200, 1800, 2400, and 3000 ms, respectively.

Section 11. Potentiation modulated by gate voltage with 5 laser pulses

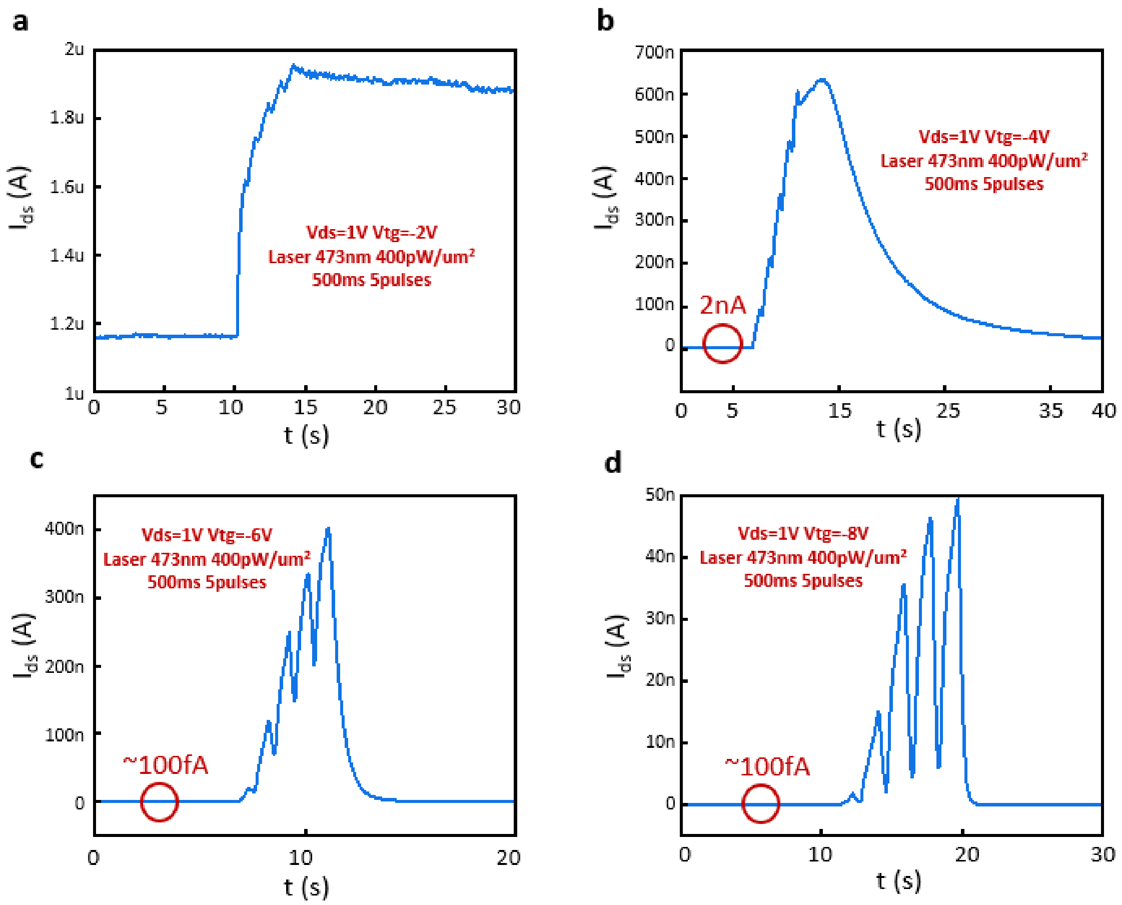

Figure S11 | The synaptic plasticity of the heterostructure device with 5 pulses at different gate voltages. a~d, response waveforms at gate voltage of -2, -4, -6, and -8 V respectively. The laser intensity is fixed at 400 pW/um^2.

Section 12. Potentiation modulated by gate voltage with 30, 50 laser pulses

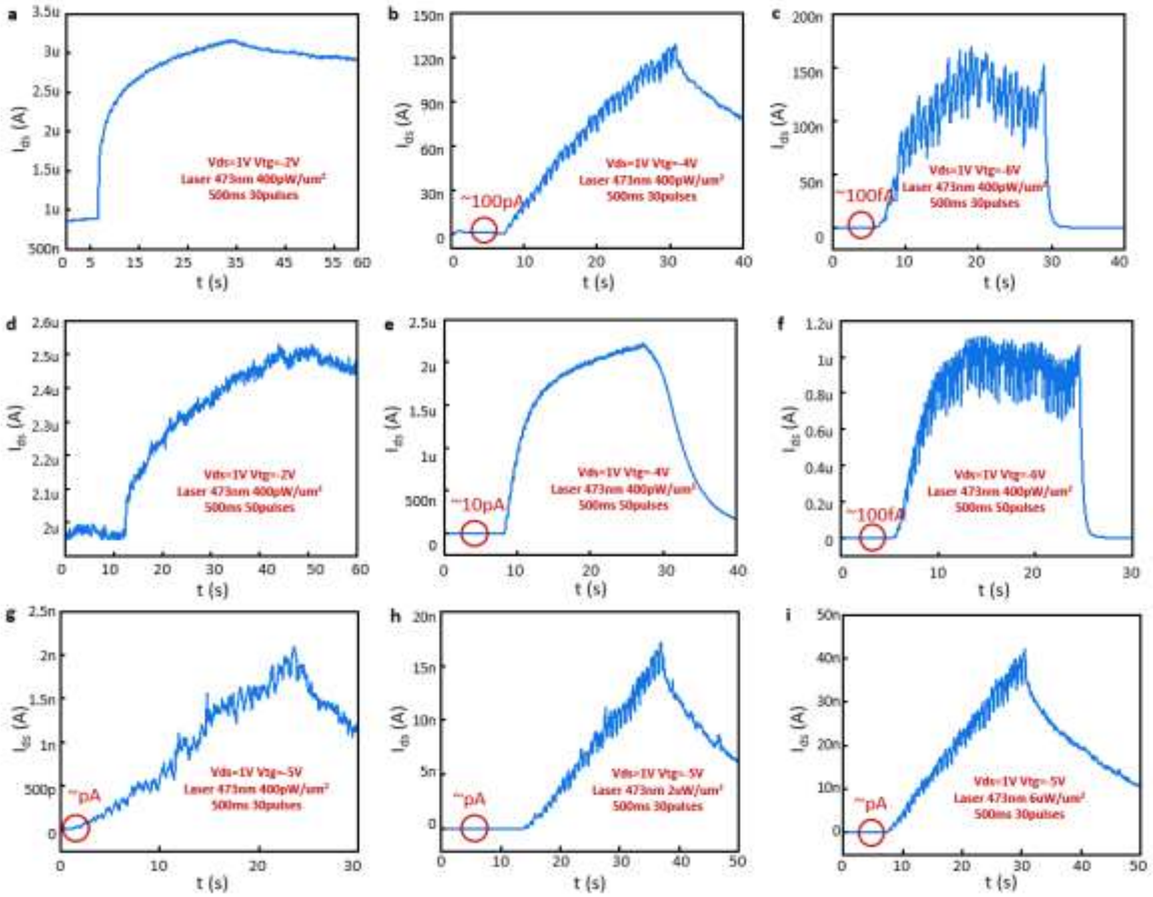

Figure S12 | The potentiation characteristics of the heterostructure synapse with 30 and 50 pulses at different conditions. a~c, LTP waveforms with 30 pulses at gate voltages of -2, -4, -6 V. **d~f**, LTP waveforms with 50 pulses at gate voltages of -2, -4, -6 V. **g~i**, LTP waveforms under different light intensities. The gate voltage is fixed at -5 V and 30 light pulses are applied. The light intensities are 400 pW/um^2, 2 uW/um^2 and 6 uW/um^2, respectively. As the negative gate voltage increases, the off-state current drops with little change in the on-state current, leading to an improvement in dynamic range. However, when the negative gate voltage goes too high, the optical response drops rapidly, affecting potentiation plasticity.

Section 13. 30 pulses long-term characteristics of VP-MoS_2 heterostructure device

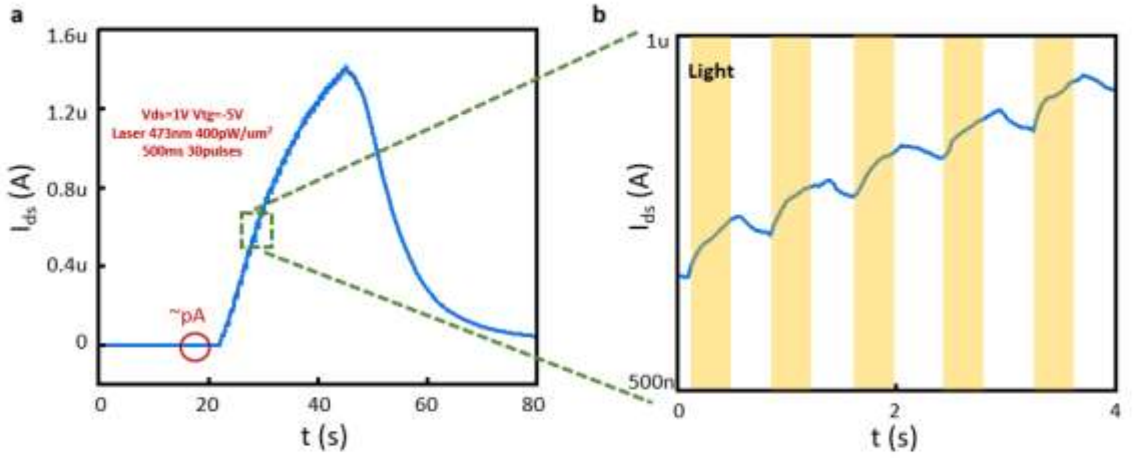

Figure S13 | 30 pulses long-term characteristics of the VP-MoS_2 device. a, Long-term characteristics of the heterostructure device. The gate voltage has been carefully selected to balance the dynamic range and linearity. The gate voltage is fixed at -5 V, and 473 nm laser pulses (500 ms illumination and 500 ms rest for each pulse) with intensities of 400 pW/um^2 have been used for stimulation. By applying 30 light pulses, the device could get 30 distinguishable conductance states, corresponding to 30 weights in neuromorphic computing. The off-state current is around pA and the on-state current could reach >1 uA, implying a high dynamic range of ~60 dB. **b,** An enlarged image for the circled part of the curve. The illumination period is shown by the yellow rectangles. The enlarged image shows distinguishable and non-crossing conductance states.

Section 14. 128 pulses long-term characteristics of VP-MoS_2 heterostructure device

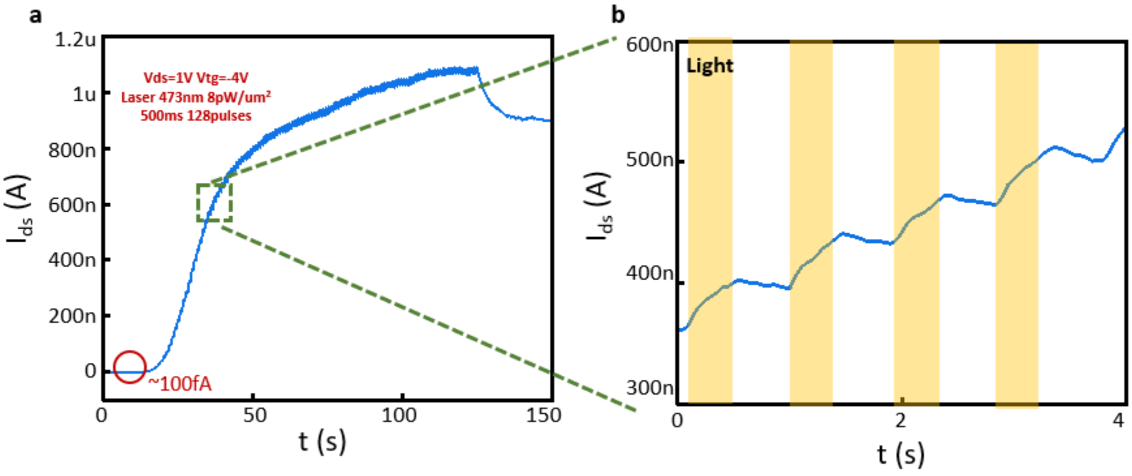

Figure S14 | 128 pulses long-term characteristics of the VP-MoS_2 device. a, Long-term characteristics of the heterostructure device. The gate voltage is fixed at -4 V, and 473 nm laser pulses (500 ms illumination and 500 ms rest for each pulse) with intensities of 8 pW/um^2 have been used for stimulation. By decreasing light intensity, more distinguishable conductance states (128 states) could be reached compared with **Figure S13**. The off-state current is around 100 fA and the on-state current could reach >1 uA, implying a high dynamic range of over 60 dB. **b,** An enlarged image for part of the curve, showing distinguishable and non-crossing conductance states.

Section 15. 200 pulses long-term characteristics of VP-MoS_2 heterostructure device

During our adjusting the light pulses, when changing the intensity and number of light pulses, the dynamic range and multi-states would change with the light pulses as well. When the light intensity and pulse number increase, the dynamic range will increase as well. However, the dynamic range could not increase infinitely. The source-drain current will gradually become saturated and will not increase with pulse number and light intensity further, with the dynamic range reach its maximum. This max dynamic range is exactly what we are concerned and used to evaluate the performance of different synaptic devices. Similarly, the multi-states would increase with the number of light pulses as well, but will finally reach a limitation when the adjacent states become crossed and undistinguishable. Specifically, when testing the multi-states performance of our device, we increase the pulse number while decreases the light intensity to get more conductance states, but always keep our dynamic reach its maximum.

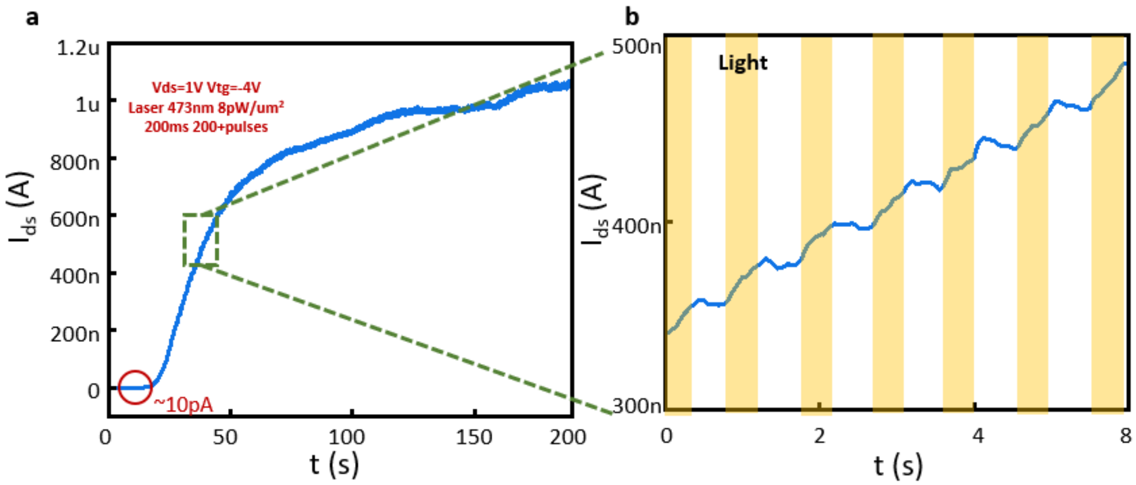

Figure S15 | 200 pulses long-term characteristics of the VP-MoS_2 device. a, Long-term characteristics of the heterostructure device. The gate voltage has been carefully selected to balance the

dynamic range and linearity. The gate voltage is fixed at -4 V, and 473 nm laser pulses (200 ms illumination and 800 ms rest for each pulse) with intensities of 8 pW/um^2 have been used for stimulation. By applying 200 light pulses, excluding a small number of crossed states, we were able to obtain a maximum of ~180 distinguishable conductance states. **b,** An enlarged image for part of the curve, showing distinguishable and non-crossing conductance states.

Section 16. Electrical excitation process of VP-MoS_2 heterostructure device

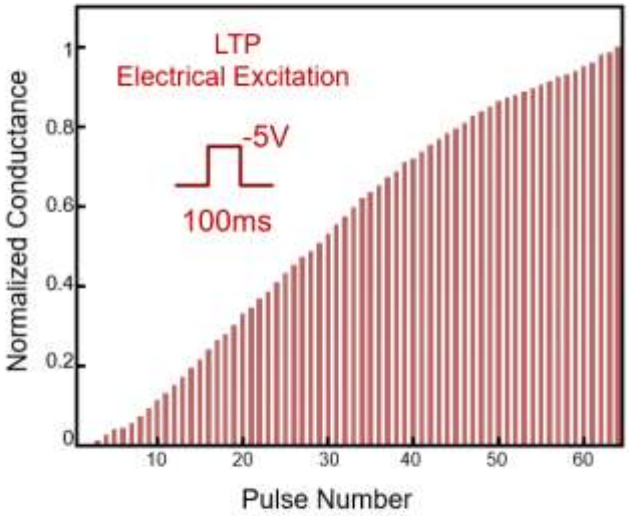

Figure S16 | Electrical long-term potential characteristics of the VP-MoS_2 device. Our device could show excitation features under both optical and electrical stimuli, and show inhabitation features only under electrical stimuli. This figure shows the LTP process (64 states) under electrical stimuli (-5V,100ms).

Section 17. Definition of distinguishable conductance states

Figure S17 a shows the detail of one conductance state extracted from **Figure S14** and **Figure S15**. After the light pulse stimulation is stopped, the source-drain current would still make small changes over time due to the instability of carrier trapping and noise. Specifically, for this conductance state, the current instability is about 12 nA. We test all the conductance states in this method for their instability and calculated the max instability is about 15 nA, which is defined as the device’s resolution, as shown in the blue shadow area in the middle and right panels. For each conductance states, if the adjacent states do not reach this area, we regard these states as distinguishable, as shown in the middle panel. On the contrast, the conductance states shown in the right panel (extracted from **Figure S15**) is undistinguishable for the shadow area of three states are crossed, and will not be counted when calculating the conductance states.

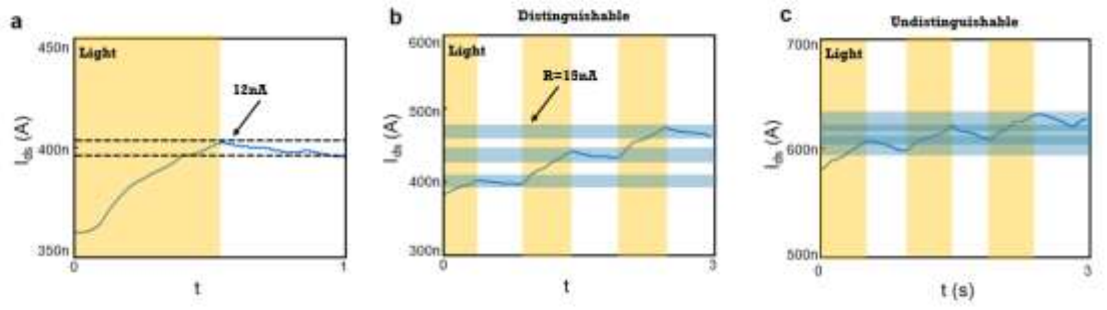

Figure S17 | Definition of distinguishable conductance states.

Section 18. Neural network simulation for image classification

Figure S18 shows a schematic of the simulation process, including the training and testing parts. The simulation is based on Neurosim simulator with a fully connected multi-layer perceptron (MLP) neural network[3]. The original simulator could only support MNIST handwriting image classification, we made necessary modifications (the number of input elements and the learning rate) to the network model to make it suitable for the Fashion-MNIST dataset.

Take the classification of the MNIST dataset as an example. First, the dataset is pre-processed into 20×20 pixels (by cropping the edges of each image) and transferred into 1-bit grey scale codes as the input elements. A network with a typical 1T1R (1 transistor with 1 resistance) structure and necessary peripheral circuits is then constructed using the VP-MoS_2 heterostructure synaptic devices. The neuron nodes of input, hidden and output layers are 400 (corresponding to the input pixels), 100, and 10 (corresponding to the output labels), separately, so the numbers of synaptic devices used in the matric W_{IH} and W_{HO} are 40k and 1k, due to the all-connected topology. Online learning is implemented in our work. In each epoch, 8k images are randomly picked from the training dataset which includes 60k images, and a testing dataset of 10k images is used to test the learning accuracy. The training process consists of two operations, the feed-forward process (FF) and the back-propagation process (BP). In the FF process, the input data travels through a series of weight sum operations (W_{IH} and W_{HO}) and neuron activation, and then reaches the output layer. After that, the result of the FF process will be compared with the label to calculate its prediction error. Then in the BP process, this error is propagated backward from the output layer to adjust the weight of each layer (W_{IH} and

W_{HO}) to minimize the error. The stochastic gradient decent method is used in the BP process. The testing process begins after 10k training sessions. The testing progress is similar to the FF process in training, which ends with the comparison with the labels without weights update. After testing 10k images and calculating the learning accuracy, here ends one epoch.

For the Fashion-MNIST dataset[4], the original dataset is 28×28 digits and is not suitable for Neurosim. Therefore, we modified the network topology of the simulator by changing the input layer to 28×28. The dataset is preprocessed into grey scale digits as well, and we also change necessary parameters in the simulation to improve the learning accuracy. However, as the original simulator is specially designed for the 20×20 MNIST dataset, the learning accuracy of the Fashion dataset is relatively lower.

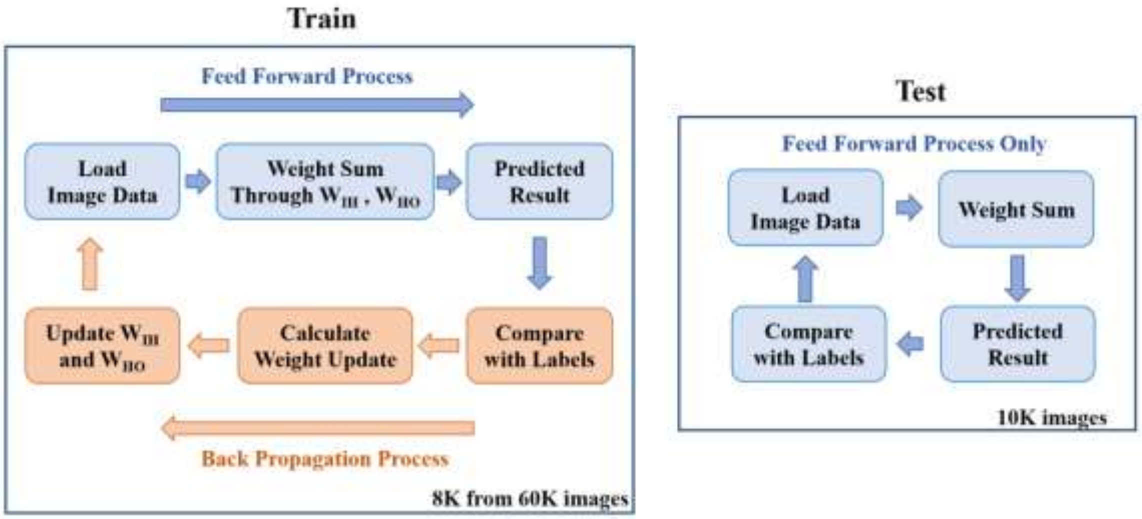

Figure S18 | A brief process of the image classification simulation.

Section 19. Conductance linearity of the VP-MoS_2 devices

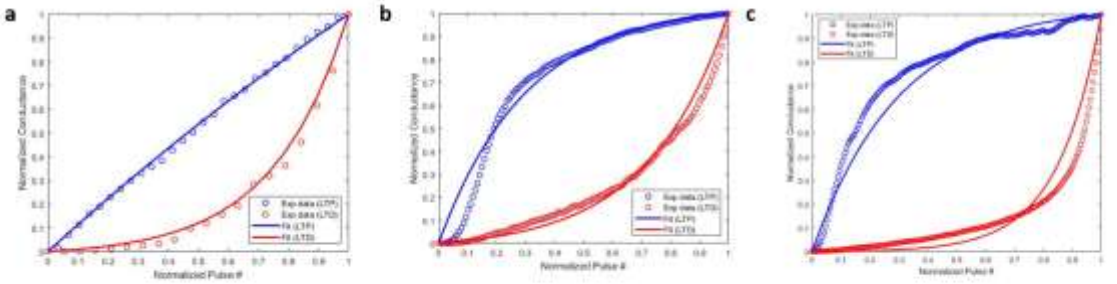

Figure S19 | The fitting result of linearity used for simulation. We use a Matlab procedure to measure the linearity of the VP-MoS_2 heterostructure device. The LTP and LTD are normalized and fitted. Inset figures a, b, and c show the results of 30 states, 128 states, and ~180 states, respectively.

Section 20. Parameters and results of the simulation

Parameters	MoS_2	VP-MoS_2	VP-MoS_2	Ideal device
Number of states	30	30	128	1000
Nonlinearity	0.38/-0.38	1.31/-2	2/-2	linear
Dynamic range	10 dB	60 dB	60 dB	∞
Resistance in 1T1R	8k	8k	8k	8k
Learning Accuracy (MNIST)	**85.09%**	**93.16%**	**95.23%**	**95.47%**
Learning Accuracy (Fashion)	**74.13%**	**77.09%**	**79.65%**	**79.95%**

Table S2 | The parameters and results in the simulation procedure. The off-state of the ideal device is set to absolute 0 to get the infinity dynamic range.

Section 21. Complexity definition of different classification tasks

In **Figure 5e**, the complexity of different image classification tasks is defined according to the average information entropy in the image dataset, which could reflect the quantity of information contained by the images. The information entropy of a single image could be defined as:

$$H(U) = -\Sigma p_i \cdot \lg(p_i)$$

Where p_i represents the distribution frequency of a certain grayscale (or RGB) value in the image. **Figure S20** shows some examples of how we calculate the complexity of different datasets. We took the image with the label "2" from three different datasets (MNIST, cropped MNIST, and Fashion), as shown in **Figure S20a, b, c**. The grayscale distribution of these three images is shown in the lower panel, respectively. According to the frequency distribution, the information entropy of these images could be calculated. It is obvious that the image from Fashion MNIST owns a more complex grayscale distribution, indicating more information it contains. It is worth mentioning that compared with the original MNIST (28×28), the cropped MNIST (20×20) owns higher information entropy, that is mainly because the cropped pixels are completely black pixels (with grayscale value 0), which contributes little to the complexity of an image but could affect the frequency of other grayscale values. As for the color images, a similar method is used, while the grayscale value is replaced with two-dimensional vectors that represent for the RGB value of a pixel.

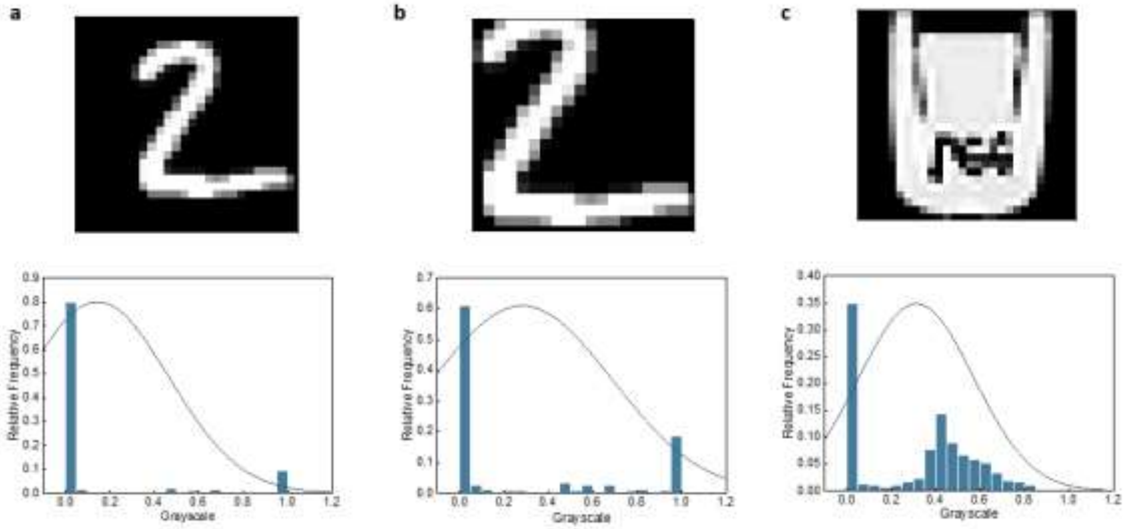

Figure S20 | Examples for the calculation of dataset complexity. Images are extracted from different dataset and calculated their information entropy according to their grayscale distribution. The average information entropy is used to define the dataset complexity.

To calculate the complexity of a dataset, we randomly picked some images with different labels and calculated their average information entropy. We defined the complexity of MNIST as 1, and calculated the relative complexity of some common datasets, as shown in **Table S3**.

Dataset	Size	Color	Complexity
QR Code	20×20	Black & White	0.36
MNIST	28×28	Black & White	1
Cropped MNIST	20×20	Black & White	1.37
Fashion	28×28	Black & White	1.66
Cifar-10	32×32	RGB	3.89
Image-net	256×256	RGB	6.21
Real Photo	1080*1440	RGB	~10.44

Table S3 | The complexity of different datasets. The original MNIST dataset is chosen as a standard to define relative complexity.

Section 22. Calculation of synaptic energy consumption

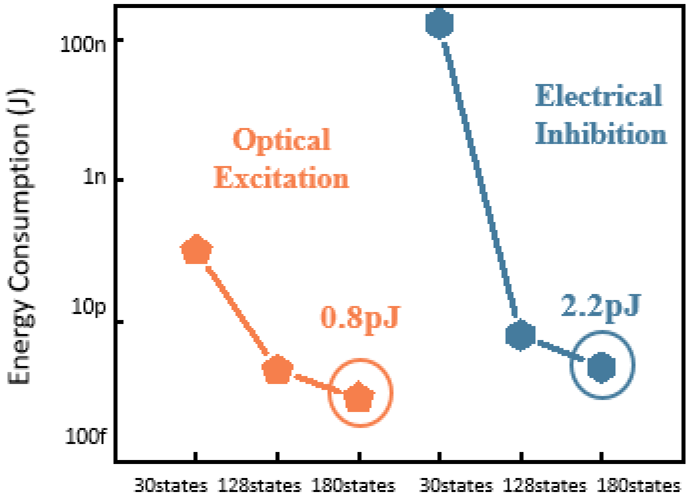

Figure S21 | Energy consumption of the synaptic device. The left and right line shows the energy consumption of optical excitation and electrical inhibition respectively.

We have calculated the energy consumption per spike for different conditions in our experiment. The electrical energy consumption is calculated according to the following equation[7]:

$$W = (V^2 \times \Delta G \times t_{pulse})/N_{pulse}$$

Where V is the training voltage, ΔG is the conductance change during training, t_{pulse} and N_{pulse} are the width and number of pulses used for training.

And the optical energy consumption is calculated according to the following equation[8]:

$$W = P_{spike} \times A_{active} \times t_{spike}$$

Where P_{spike} is the spike power, A_{active} is the active area, t_{spike} is the spike width.

The results of our calculation are shown in **Figure S21**. The left and right panel shows the energy consumption for optical excitation and electrical inhibition, respectively. The lowest energy consumption of our synaptic devices is 0.8pJ per spike, which is comparable to the consumption of the human brain.

References:

1 Zhang L. H., et al. Structure and properties of violet phosphorus and its phosphorene exfoliation. *Angew. Chem. -Int. Edit.* **59**, 1074-1080 (2020)

2 Baumer F., et al. Synthesis, Characterization, and device application of antimony-substituted violet phosphorus: a layered material. *Acs Nano* **11**, 4105-4113 (2017)

3 Zhao R. Z., et al. Violet phosphorus quantum dots. *J. Mater. Chem. A* **10**, 245-250 (2021)

4 Mak K. F., Lee C., Hone J., Shan J.&Heinz T. F. Atomically thin MoS_2: a new direct-gap semiconductor. *Phys. Rev. Lett.* **105**, (2010)

5 Chen P. Y., Peng X. C.&Yu S. M. NeuroSim plus : an integrated device-to-algorithm framework for benchmarking synaptic devices and array architectures. *in 2020 IEEE International Electron Devices Meeting (IEDM)* 6.1.1-6.1.4 (2017)

6 Xiao, H., Rasul, K., & Vollgraf, R. Fashion-MNIST: a novel image dataset for benchmarking machine learning algorithms. *ArXiv*, abs/1708.07747. (2017)

7 Wang, T. Y. et al. Ultralow Power Wearable Heterosynapse with Photoelectric Synergistic Modulation. Adv Sci 7, doi:ARTN 1903480 10.1002/advs.201903480 (2020).

8 Zhu, C. G. et al. Optical synaptic devices with ultra-low power consumption for neuromorphic computing. Light-Sci Appl 11, doi:ARTN 337 10.1038/s41377-022-01031-z (2022).
